# Antiemetic effect of acupressure wristbands for GLP-1 medication associated nausea

**DOI:** 10.1016/j.obpill.2025.100178

**Published:** 2025-05-08

**Authors:** Florencia Ziemke, Soufiane Belarj, Jem Esguerra, Anita Reyes, Nawfal Istfan

**Affiliations:** a Evexia Medical, Jupiter, FL, USA; b BellaVida Family Practice, Royal Palm Beach, FL, USA; c Brigham and Women’s Hospital, Harvard Medical School, Boston, MA, USA

**Keywords:** Acupressure, Drug-free, Glp1, Glp-1 medication, Nausea, Nausea relief

## Abstract

**Background:**

Nausea is one of the most reported side effect of GLP-1 receptor agonists (GLP-1a). Current recommendations fall short in taming the symptoms, include antiemetic medication, behavior changes, GLP-1a dose adjustment, and often cause a disruption to treatment. Sea-Band® is a drug-free, class II FDA-cleared medical device for relief of nausea in motion sickness, morning sickness, chemotherapy and anesthesia induced nausea. The device is a set of soft, elastic, reusable acupressure wristbands (ACW) with a skin-facing plastic button worn below the wrist crease applying pressure at acupoint pericardium 6. We hypothesized that ACW was an effective tool for GLP-1a associated nausea.

**Methods:**

This was a one-arm, open-label, non-randomized, prospective interventional study evaluating the antiemetic effect of ACW in non-pregnant adults on GLP-1as with nausea. GLP-1a were semaglutide or tirzepatide. Exclusion criteria were patients on GLP-1a without nausea, recent use of antiemetic medications, other nausea-related conditions, history of gastroparesis, and uncontrolled gastroesophageal reflux disease. Patients were shown how to properly place and use ACW at the onset of nausea and were followed weekly for 4 weeks. Follow-ups assessed frequency of nausea, ACW use frequency and duration, and change in nausea.

**Results:**

359 episodes of nausea were recorded amongst 31 adult participants over 4 weeks. Adults, mean age 55, mean BMI 34, mean HbA1c 5.9 %, reported nausea over 80 % of the time on a weekly basis. ACW were used in all recorded episodes of nausea. Medication doses were kept stable throughout the duration of this study. Nausea relief was achieved within 5 min in one third of episodes, and in over 5 min but under 20 min in the remainder of the episodes. A logistic regression model was used to evaluate the likelihood of nausea relief. A consistent rate of nausea relief over 80 % was observed during the study period, adjusting for the correlation between reduced nausea episodes and reduced episodes.

**Conclusion:**

Although not a controlled trial, this pilot, proof of concept, pragmatic study suggests that ACW may offer a safe, self-administered, reusable, and drug-free option for managing GLP-1a associated nausea.ACW’s nausea reducing effect was seen in over 80 % of episodes, and remained consistent throughout the study period. One third of participants experienced relief within 5 min of wearing ACW in the first three weeks. Given the relatively small sample size of the population, further large-scale investigations are justified. Nausea is common in day-to-day real-world use of GLP-1as, and our results suggest that using ACW may provide a first-line therapeutic intervention used ad libitum to tame a disruptive symptom, improve day-to-day well-being, and positively impact a person’s treatment journey on GLP-1a.

## Graphical abstract

**Figure:**
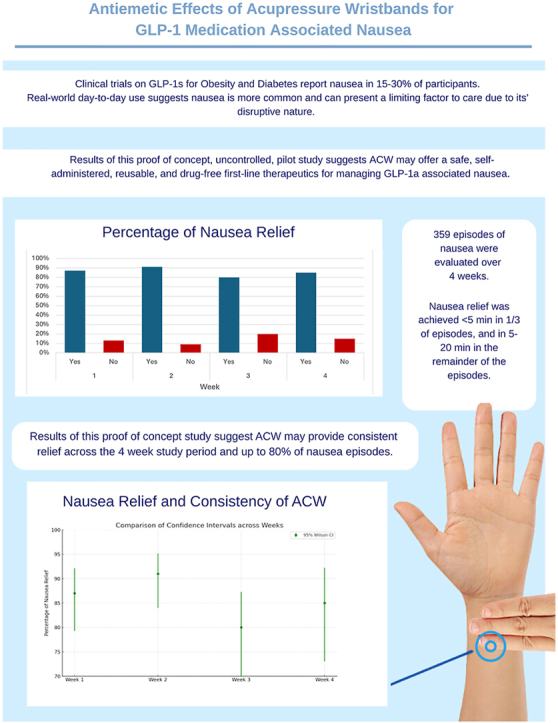

## 1 Introduction

Obesity is a global leading non-communicable disease (NCD) due to its significant impact on health and association with numerous chronic conditions including Type 2 diabetes [1,2]. Clinical treatment guidelines consider pharmacotherapy use in adjunct to lifestyle and dietary interventions [3,4]. A class of drugs collectively known as Glucagon-like peptide-1 receptor agonists (GLP-1a) are recognized in clinical practice for the treatment of Type 2 diabetes, Obesity or Overweight with weight related comorbidities. [5]. The most common adverse effects of these agents are gastrointestinal, namely nausea and constipation, and often represent a therapeutic barrier to their use in clinical practice, despite their established health benefits [[6], [7], [8], [9], [10]]. Clinical trials on GLP-1a suggest the incidence of nausea ranges from 15 to 30 % of study participants [[6], [7], [8]] yet day to day clinical use shows that nausea has greater variability amongst users. Current recommendations for GLP-1a nausea relief includes short-term use of anti-nausea medication, adjusting the dose of the GLP-1a, and dietary changes such as reducing the meal size, stopping to eat when feeling full, avoiding high-fat or spicy foods and moderating alcohol consumption [11]. Nausea remains a common cause of discomfort in persons taking GLP-1a and current recommendations for nausea management fall short.

Acupressure is a non-invasive, traditional Chinese technique that substitutes acupuncture needling by applying skin pressure [12]. The acupoint most commonly used to achieve antiemesis is pericardium point 6 (P6), also known as the Nei Kuan point [13]. The P6 acupoint is located on the volar surface of the forearm, approximately three finger-breadths proximal to the wrist crease between the tendons of flexor carpi radialis and palmaris longus muscles, at approximately 1 cm depth. Numerous clinical trials, including randomized placebo-controlled studies, have established P6 acupressure for nausea relief [[14], [15], [16], [17]]. A class II FDA cleared medical device is a set of drug-free, elastic acupressure wristbands (ACW) with a skin facing plastic nodule designed to apply pressure at the P6 point at both wrists. This device is used for nausea relief in motion sickness [18,19], morning sickness in pregnancy [[20], [21], [22], [23]], post-operative nausea [17,[24], [25], [26], [27], [28], [29]] and chemotherapy related nausea [13,[30], [31], [32], [33], [34], [35], [36], [37]]. Previous clinical trials [13,21,34,36,38] have established its’ effectiveness for nausea relief for other indications, and four decades of real-world use suggest nausea relief is traditionally seen within 5 min.

Nausea related to GLP-1a is believed to arise from direct effects on the central nervous system, mediated by GLP-1 receptors in the area postrema, a highly vascular paired structure in the medulla oblongata in the brainstem [9]. The effect of GLP-1a on gastrointestinal (GI) function, namely delaying of gastric emptying and intestinal motility, additionally contribute to the occurrence of GI side effects as a whole [9,39,40]. Use of prescription anti-nausea medication can present a challenge with drug interactions if a person is on other medications, and these anti-emetics are not side effect free. Adjusting the dose of GLP-1a may not always align with therapeutic goals of the user. Dietary changes alone are easier said than done and may not be sufficient to tame the symptom.

We hypothesized that ACW could alleviate GLP-1a induced nausea in adults actively taking these medications. To our knowledge, this is the first evaluation of ACW for the relief of nausea associated with the use of GLP-1as. The primary outcome was change in nausea after wearing ACW to assess the antiemetic effect in adults taking GLP-1a. Secondary outcomes included time to nausea relief in under five (5) minutes, or over five (5) minutes.

## 2 Methods

This was a one-arm, open-label, non-randomized, prospective interventional study evaluating the antiemetic effect of ACW in non-pregnant adults currently on GLP-1a, who presented to their routine follow up appointments with nausea. Institutional Review Board (IRB) review and approval was obtained for this study. Informed consent was obtained from all participants prior to their inclusion in the study. Participants were provided with a detailed information sheet explaining the purpose of the study, the procedures involved, the potential risks and benefits, and their rights as participants, including the right to withdraw at any time without consequence. The consent process ensured that participants had sufficient time to ask questions and were given the opportunity to review the consent form. GLP-1as used in this study included semaglutide and tirzepatide Exclusion criteria for participation were patients on GLP-1as without nausea, recent use of antiemetic medications, other nausea-related conditions such as vertigo, history of gastroparesis, and uncontrolled gastroesophageal reflux disease. ACW used for this study was Sea-Band®, which are a pair of knit, soft, elastic, reusable and washable wristbands and a FDA-cleared class II medical device. Upon enrollment in the study, participants were given a set of ACW and shown how to properly place the elastic bands at the P6 acupoint, located at three fingerbreadths below the crease of each wrist. Participants were given a chance to practice correctly placing ACW. The demonstration of proper placement took approximately two (2) minutes per participant. They were instructed to wear ACW at the onset of nausea, record whether ACW nausea relief, and include the time to relief (less than 5 min versus more than 5 min). Demographic information and baseline characteristics were captured at the start of the study. Questionnaires were used to collect weekly data for a total of four weeks.

## 3 Results

### 3.1 Study sample baseline descriptive characteristics

A total of three hundred and fifty-nine (359) episodes of nausea were reported in thirty-one (31) adults over the course of four (4) weeks. Participants age ranged from 24 to 80 years, with a median age of 55 and a variability of 15 years, indicating a moderately diverse age range (Table 1). While most participants were middle-aged or older, there was a noticeable skew toward the younger end of the spectrum. Study participant Body Mass Index (BMI) varied from 29 to 63 kg/m^2^ with a mean of 35, and a standard deviation of 6.6. A higher percentage of participants had lower BMI values within the entire range, as indicated by the BMI histogram's modest right skew. A certain amount of variability is highlighted by the skewness seen in the age and BMI distributions, implying that the study group was not evenly distributed across these measures. (Table 1).

**Table 1:** Descriptive statistics: Age, BMI (m/kg^2^).

Metric/Variable	Age	BMI
Min	24	29
Max	80	63
Average	55	35
Median	55	34
Standard deviation	15	6.6

Thirty-two (32) percent of participants were men and 68 % women. The majority (71 %) identified as White (W), followed by African American (AA) at 19 % and Hispanic (H) at 10 %. The study group’s medication was semaglutide (58 %), and tirzepatide (42 %)(Table 2). The mean HbA1c level was 5.97 %, with a median HbA1c is 5.9 %, with a 95 % confidence interval ranging from 5.86 to 6.08. which closely aligns with the mean, suggesting a relatively symmetrical distribution without significant skewness. To further understand the reliability of the mean, a 95 % confidence interval was calculated, ranging from approximately 5.86 to 6.08.

**Table 2:** Descriptive statistics: Gender, race, medication and HbA1c (%).

Variable	Frequency	Distribution
Gender		
Male	10	30 %
Female	21	70 %
Race		
White	22	70 %
AA	6	20 %
Hispanic	3	10 %
Medication		
Tirzepatide	13	40 %
Semaglutide	18	60 %
HbA1c (%)	Mean 5.97 (95 % CI 5.9–6.1)Min 5.5, Max 6.7	

### 3.2 Nausea episodes

Participants were asked to record self-reported nausea episodes over a period of four (4) weeks. The average number of nausea episodes per week was variable and in general decreased over time. (Table 3). The per person distribution in the occurrence of nausea during the study period showed an average of 11.13, SD = 5.11. (Table 3).

**Table 3:** Episodes of nausea per week and per person distribution over four weeks.

Variable	Frequency	Distribution
Episodes of Nausea		
Week 1	105	
Week 2	106	
Week 3	81	
Week 4	53	
Per Person Episode of Nausea over 4 Weeks		
Average	11.13	Min 2
SD	5.11	Max 23

**Graph 1:**
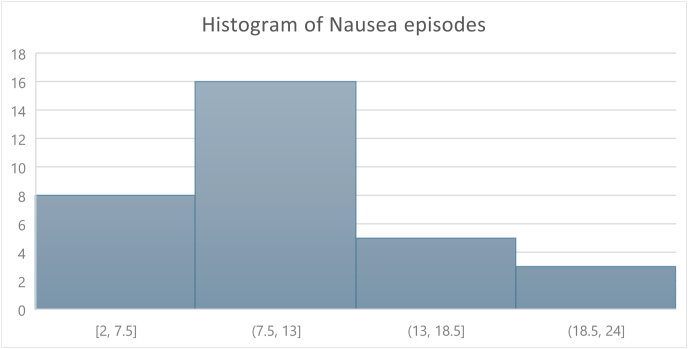
Histogram of Nausea episodes per person per Study period of 4 weeks.

When determining the histogram of nausea episodes per person over the study period, the (7.5, 13] bin dominates, representing individuals clustering near the mean of 11.13., which is the largest group in the histogram. (Graph 1). The standard deviation of 5.11 indicates moderate variability, meaning some individuals experienced nausea episodes far from the mean. The data appears slightly skewed to the right, as there are more individuals with values in the lower-to-moderate range ((2, 7.5] and (7.5, 13]) and fewer in the higher ranges ((13, 18.5] and (18.5, 24]). This distribution suggests that nausea episodes are relatively evenly spread across individuals but are concentrated around the average, ensuring nausea episodes are not overly concentrated in only a few individuals. Additionally, this study evaluated nausea relief per se, independent of the medication type, dose, nor stage of treatment. Doses of semaglutide were 0.25 mg and 0.5 mg. Doses of tirzepatide were 2.5 mg and 5.0 mg. The occurrence of nausea on specific days of the week, relative to the day of medication administration was not specifically analyzed in this study.

### 3.3 Nausea relief and timing of Nausea relief using ACW

**Graph 2:**
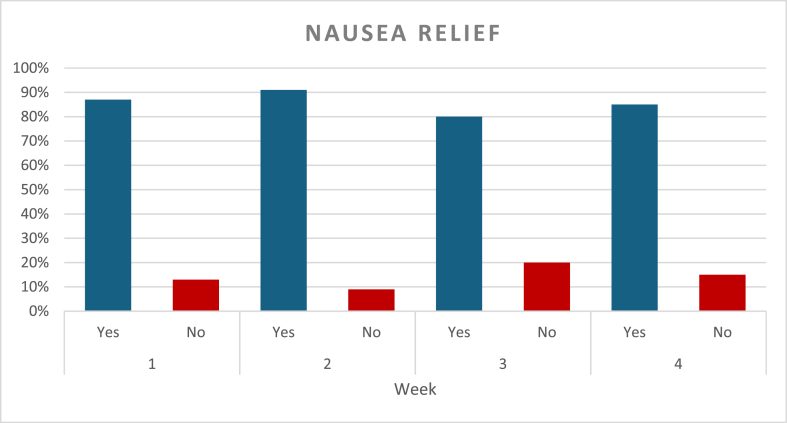
Percentage of Nausea relief over the course of four weeks.

**Graph 3:**
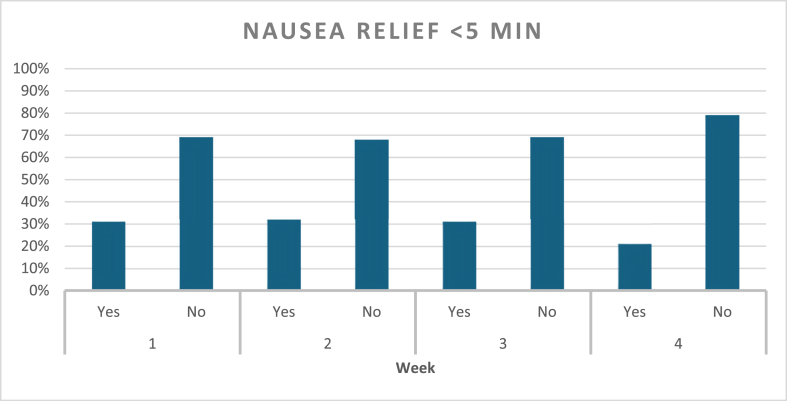
Percentage of Nausea relief within five minutes of ACW use.

Nausea episodes and ACW response were assessed on a weekly basis (Graph 2). During the first week, a total of 105 nausea episodes were reported, accounting for 98 % of participants, and all of them used ACW. Relief was achieved in 87 % of these episodes, with 31 % resulting in relief within 5 min of applying ACW, and 69 % requiring more than 5 min to relief, but less than 20 min. In the second week, all participants had nausea, and a total of 106 episodes were reported, with wristbands being used in all cases. Relief was obtained in 91 % of episodes, with 32 % of the relief occurring within 5 min, and 68 % requiring longer use of the bands. By the third week, nausea episodes dropped overall, and a total of eighty-one episodes were reported. Relief occurred within 5 min for 31 % of episodes, while 69 % took longer than 5 min and under 20 min for relief. During the fourth week follow up, a total of fifty-three episodes of nausea were reported showing an overall decline compared to the previous weeks. ACW were used in every episode. Relief was reported in 85 % of episodes, with 21 % experiencing nausea relief within 5 min and 79 % of users taking longer than 5 min to experience relief (Graph 3). Study participants highlighted the ease of ACW use, and no adverse reactions were reported during the four-week duration of this study.

### 3.4 Consistency of Nausea relief with ACW over time

**Graph 4:**
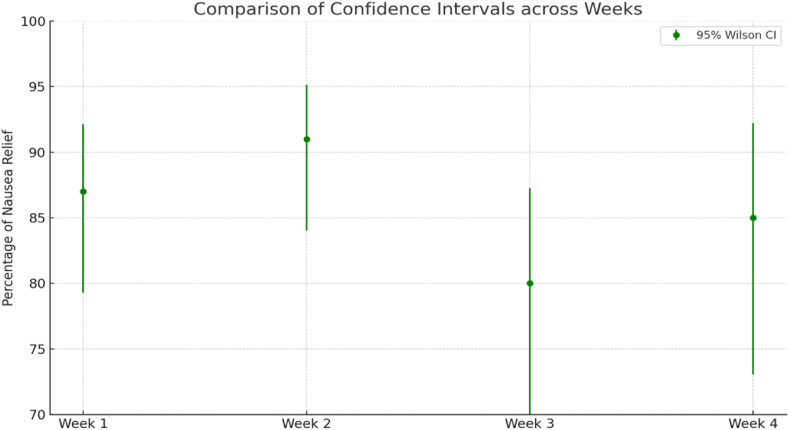
Nausea Relief and Consistency of ACW. Percentage of Nausea relief versus time.

This study followed the same group of participants over a four-week period evaluating nausea relief with ACW in self-recorded episodes of nausea. The number of nausea episodes decreased progressively from 105 in Week 1–53 in Week 4. (Graph 4). Results show that the 95 % confidence intervals for the observed nausea rates increased over the study period. Specifically, the margin between the upper bound and the observed rate of nausea: week 1, 5 %, week 2, 4 %, week 3, 7 %, week 4, 7 %. The widening confidence intervals are attributed to a decrease in sample size, driven by a lower number of self-reported nausea episodes. Based on these findings, we observe a stable 87 % relief rate throughout all weeks while adjusting for the decrease in sample size of self-reported nausea episodes and demonstrate that ACW maintained consistent effectiveness in providing nausea relief.

### 3.5 Logistic regression analysis for Nausea relief

A logistic regression model was used to evaluate the likelihood of nausea relief (Yes/No) across Weeks 1–4, with Week 4 as the reference category. The coefficients (β), significance levels (p-values), and odds ratios (OR) are summarized below (Table 4).

**Table 4:** Logistic regression to evaluate likelihood of nausea relief.

Week	Coefficient (β)	Significance (p-value)	Odds Ratio (OR)	Interpretation
Week 1	0.23	0.239	1.258	No significant effect.
Week 2	0.535	0.08	1.707	Marginal positive effect.
Week 3	−0.325	0.0792	0.722	Marginal negative effect.
Week 4	Reference	–	1.00	Baseline comparison.

## 4 Discussion

Results of this uncontrolled, pilot, proof of concept study suggest that ACW may provide consistent relief across the study period and up to 80 % of nausea episodes.

The purpose of this study was to assess nausea relief using a drug-free, over the counter, self-administered, reusable device, in adults on GLP-1a presenting with nausea. Placebo-controlled trials have well established the use of ACW for other nausea related indications such as postoperative nausea, motion sickness, morning sickness in pregnancy and chemotherapy associated nausea in persons aged three years and older. This proof-of-concept study specifically evaluated the use of ACW in adults with GLP-1a associated nausea.

Detailed logistic regression analysis further underscores variations in nausea relief across the weeks. Week 2 was associated with a marginally significant positive effect on nausea relief, as evidenced by a higher likelihood of symptom improvement during this period (91 % of episodes showing improvement). Conversely, Week 3 displayed a marginally negative effect, with a lower percentage of rapid relief (31 % of episodes within 5 min) compared to other weeks. These fluctuations could reflect individual differences in response to ACW, variations in episode severity, or other external factors influencing outcomes.

Participant highlighted the practicality of a self-administered, easy to apply, reusable tool to tame their symptoms. Instructions on how to use ACW and how to properly place them at the three fingerbreadths mark below the crease of each wrist, took approximately two (2) minutes per participant.

One interesting finding is the timing of symptoms relief. We included a time to relief of under 5 min after wearing ACW in the analysis based on four decades of real-world use of these bands for other indications. A larger portion of study participants noted nausea relief after more than 5 min and mostly within 15–20 min, specifically, 69 % in Week 1, 68 % in Week 2, and 79 % in Week 4. Nausea relief is perhaps more gradual in this group compared to other indications and warrants future studies.

## 5 Limitations

Future research should prioritize studies to address these limitations. Such studies could also explore ACW performance across diverse patient populations, explore nausea relief comparison of drug types, i.e. semaglutide versus tirzepatide, consider the cumulative relief of acupressure over time and its’ effect on nausea relief, and identify factors that may influence individual responses. By building on the findings of this study, future investigations will help solidify the role of ACW as a first-line therapeutic intervention for GLP-1a-associated nausea.

## 6 Conclusion

Obesity and Diabetes are the twin epidemics of the 21st century, and numbers continue to escalate at a dramatic rate, which is a call to action. GLP-1as are revolutionizing the approach to improvement in health when used for these diseases, but nausea remains a common complaint. Clinical trials reports an incidence of nausea in 15–30 % of GLP-1a users, however real-world use of these medications shows greater variability.

From the patient perspective, the challenge is that nausea is an unpleasant experience, subjectively described as an *uneasy sickness to the stomach,* that can disrupt day to day activities and quality of life. Current recommendations to relieve nausea associated with GLP-1as include prescribing anti-emetics, which are not side-effect free. Other recommendations include dietary and lifestyle changes such as avoiding fatty foods, eating small portions which are easier said than done a may not sufficiently tame the symptom of nausea.

We evaluated the antiemetic effect of ACW in three hundred and fifty-nine cases of nausea over a period of four weeks in adults on GLP-1a. Although not a controlled trial, this proof of concept, pragmatic study suggests that ACW may offer a safe, self-administered, reusable, drug-free and first-line therapeutic option for managing GLP-1a–associated nausea. Given the small sample size, further larger-scale, controlled studies may provide more definitive insight to the clinical application of ACW for this indication.

Take away Points:

• Nausea related to GLP-1a often presents a limiting factor to care due to its' disruptive nature. ACW is a drug-free device that can be self-administered, reused, and serve as a tool in managing GLP-1a related nausea.
• In this uncontrolled, pilot, proof of concept study, ACW provided consistent relief for over eight (80) % of nausea episodes across the four-week study period in adults taking GLP-1a. Controlled follow-up studies are justified to confirm these findings.
• The time to relief when using ACW varied among users; while the majority took over 5 min and under 20 min, about 1/3 of nausea episodes responded to ACW within 5 min during the initial 3 weeks.

## Dualities of interest

Florencia Ziemke is on the Editorial Board of Obesity Pillars, serves on the Advisory Board for Novo Nordisk, Speaker’s bureau for Novo Nordisk, Speaker’s bureau Eli Lilly and Co, a Medical consultant for Nestle Health Sciences, and Sea-Band Ltd.

Jem Esguerra, Soufiane Belarj, Anita Reyes have no potential conflicts of interest.

Nawfal Istfan has no potential conflict of interest.

## Ethical review section

This was an uncontrolled prospective, interventional, proof of concept pilot study.

## Author contribution

The concept of the submission was by Florencia Ziemke, Soufiane Belarj and Nawfal Istfan, Anita Reyes participated as an investigator in the study and edited the draft, Jem Esguerra reviewed and edited the draft, Soufiane Belarj performed the statistical analysis, Nawfal Istfan proposed the statistical analysis, wrote, reviewed and edited the results and publication. Florencia Ziemke wrote, reviewed and approved the final submission and publication.

## Author contribution

The concept of the submission was by Florencia Ziemke, Soufiane Belarj and Nawfal Istfan, Anita Reyes participated as an investigator in the study and edited the draft, Jem Esguerra reviewed and edited the draft, Soufiane Belarj performed the statistical analysis, Nawfal Istfan proposed the statistical analysis, reviewed and edited the results and publication. Florencia Ziemke wrote, reviewed and approved the final submission and publication.

## Disclosures

Florencia Ziemke is a medical advisor and consultant for Novo Nordisk, Eli Lilly, Sea-Band Ltd, Nestle Health Sciences.

## Declaration of artificial intelligence (AI) and AI-assisted technologies

During the preparation of this work the author(s) did not use AI.

## Funding

This research is investigator-initiated. Sea-Band® Ltd provided the wristbands used in this study. No other specific funding was received for this investigation.
