# Supplementary material for: Antiemetic effect of acupressure wristbands for GLP-1 medication associated nausea
Source: Obes Pillars. 2025 May 8;15:100178. doi: 10.1016/j.obpill.2025.100178 (PMC12137197; doi:10.1016/j.obpill.2025.100178)

**Supplemental Material**

1. **Tables**

**Table 1: Descriptive Statistics: Age, BMI (m/kg^2^)**

| Metric/Variable | Age | BMI |
| --- | --- | --- |
| Min | 24 | 29 |
| Max | 80 | 63 |
| Average | 55 | 35 |
| Median | 55 | 34 |
| Standard deviation | 15 | 6.6 |

**Table 2: Descriptive Statistics: Gender, Race, Medication and HbA1c (%)**

| Variable | Frequency | Distribution |
| --- | --- | --- |
| Gender | | |
| Male | 10 | 30% |
| Female | 21 | 70% |
| Race | | |
| White | 22 | 70% |
| AA | 6 | 20% |
| Hispanic | 3 | 10% |
| Medication | | |
| Tirzepatide | 13 | 40% |
| Semaglutide | 18 | 60% |
| HbA1c (%) | Mean 5.97 (95% CI 5.9-6.1)  Min 5.5, Max 6.7 |  |

**Table 3. Episodes of Nausea per Week and per Person Distribution over Four Weeks.**

| Variable | Frequency | Distribution |
| --- | --- | --- |
| Episodes of Nausea | | |
| Week 1 | 105 |  |
| Week 2 | 106 |  |
| Week 3 | 81 |  |
| Week 4 | 53 |  |
| Per Person Episode of Nausea over 4 Weeks | | |
| Average | 11.13 | Min 2 |
| SD | 5.11 | Max 23 |

**Table 4. Logistic Regression to Evaluate Likelihood of Nausea Relief.**

| Week | Coefficient (β) | Significance (p-value) | Odds Ratio (OR) | Interpretation |
| --- | --- | --- | --- | --- |
| Week 1 | 0.23 | 0.239 | 1.258 | No significant effect. |
| Week 2 | 0.535 | 0.08 | 1.707 | Marginal positive effect. |
| Week 3 | -0.325 | 0.0792 | 0.722 | Marginal negative effect. |
| Week 4 | Reference | — | 1.00 | Baseline comparison. |

1. **Graphs**

**Graph 1: Histogram of Nausea episodes per person per Study period of 4 weeks**


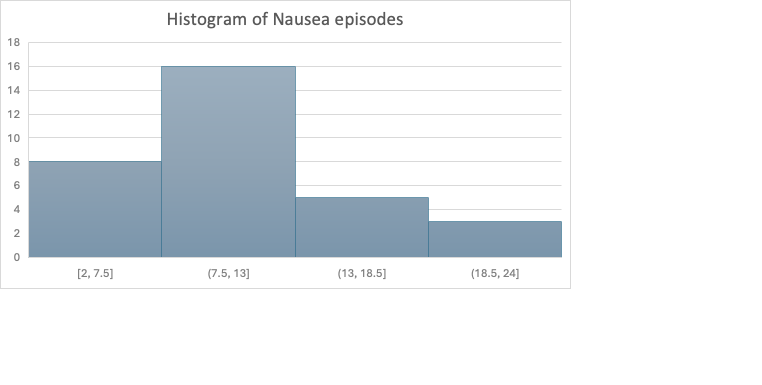


**Graph 2: Percentage of Nausea Relief over the Course of Four Weeks**

**Graph 3: Percentage of Nausea Relief within Five Minutes of ACW Use**

**Graph 4: Nausea Relief and Consistency of ACW. Percentage of Nausea relief versus time.**


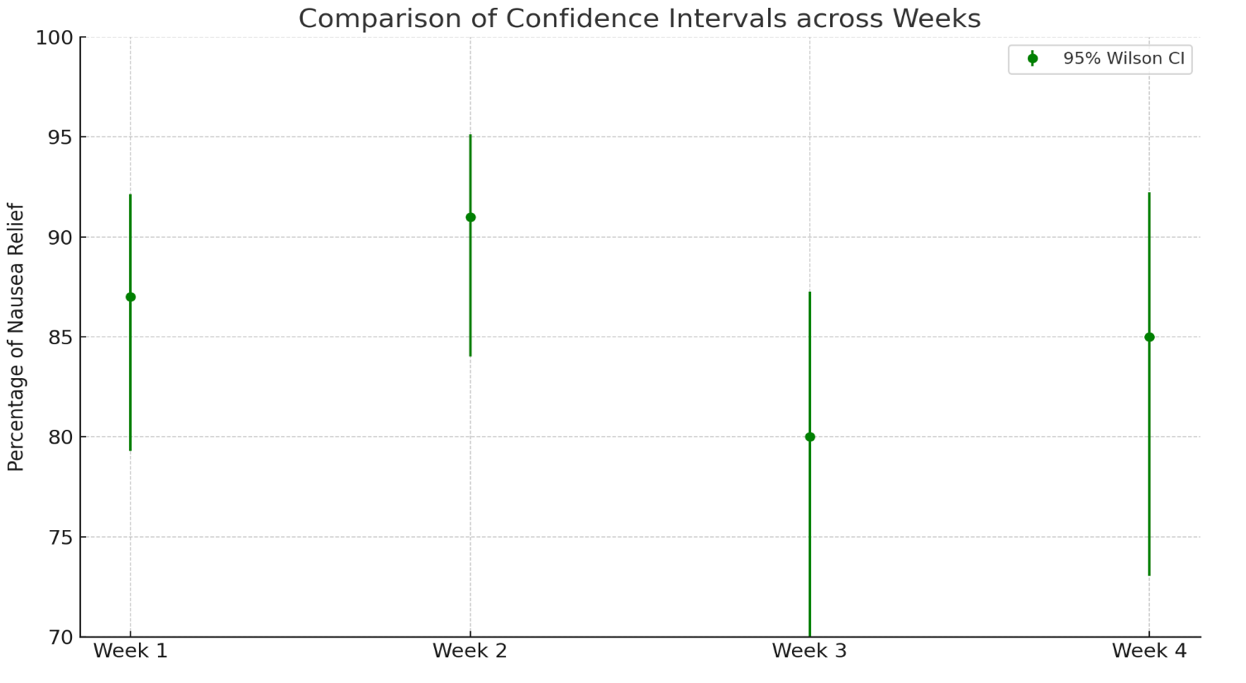

Supplement: Multimedia component 1 [file mmc1.docx]
